# Resilience: Safety in the Aftermath of Traumatic Stressor Experiences

**DOI:** 10.3389/fnbeh.2020.596919

**Published:** 2020-12-21

**Authors:** Kimberly Matheson, Ajani Asokumar, Hymie Anisman

**Affiliations:** ^1^Department of Neuroscience, Carleton University, Ottawa, ON, Canada; ^2^The Royal Ottawa's Institute of Mental Health Research, Ottawa, ON, Canada

**Keywords:** resilience, traumatic stressors, biopsychosocial, safety net, post-trauma, social connection, spirituality, nature

## Abstract

The relationship between adverse experiences and the emergence of pathology has often focused on characteristics of the stressor or of the individual (stressor appraisals, coping strategies). These features are thought to influence multiple biological processes that favor the development of mental and physical illnesses. Less often has attention focused on the aftermath of traumatic experiences, and the importance of safety and reassurance that is necessary for longer-term well-being. In some cases (e.g., post-traumatic stress disorder) this may be reflected by a failure of fear extinction, whereas in other instances (e.g., historical trauma), the uncertainty about the future might foster continued anxiety. In essence, the question becomes one of how individuals attain feelings of safety when it is fully understood that the world is not necessarily a safe place, uncertainties abound, and feelings of agency are often illusory. We consider how individuals acquire resilience in the aftermath of traumatic and chronic stressors. In this respect, we review characteristics of stressors that may trigger particular biological and behavioral coping responses, as well as factors that undermine their efficacy. To this end, we explore stressor dynamics and social processes that foster resilience in response to specific traumatic, chronic, and uncontrollable stressor contexts (intimate partner abuse; refugee migration; collective historical trauma). We point to resilience factors that may comprise neurobiological changes, such as those related to various stressor-provoked hormones, neurotrophins, inflammatory immune, microbial, and epigenetic processes. These behavioral and biological stress responses may influence, and be influenced by, feelings of safety that come about through relationships with others, spiritual and place-based connections.

## Introduction

Research concerning the impacts of stressors has long focused on determining underlying biopsychosocial processes and how these might give rise to various psychological and physical pathologies. Numerous experiential and individual difference factors (e.g., gender, ethnoracial status, age, earlier stressor encounters) that could exacerbate or diminish the actions of stressors have been established (Anisman et al., 2018). Likewise, genetic and epigenetic factors that code for particular biological features (e.g., processes related to glucocorticoid or neurotrophin functioning) are tied to the occurrence of behavioral and physical disturbances (Suri et al., 2013; Szyf et al., 2016; Mehta et al., 2020), and glucocorticoids influence epigenetic responses to stress (Reul et al., 2014). These actions are further affected by the type and timing of stressor challenges (Burns et al., 2018; Torres-Berrío et al., 2019). However, there are so many experiential and biological (hormonal, neurochemical, growth factor, and inflammatory) changes associated with stressful events that it may be impractical to assign a given process to any particular pathology. Instead, it may be more productive to identify *signatures* that comprise a constellation of factors (much as in a precision medicine approach) that might account for specific features of various pathologies, and vulnerability or resilience regarding their development and progression.

**Figure 1:**
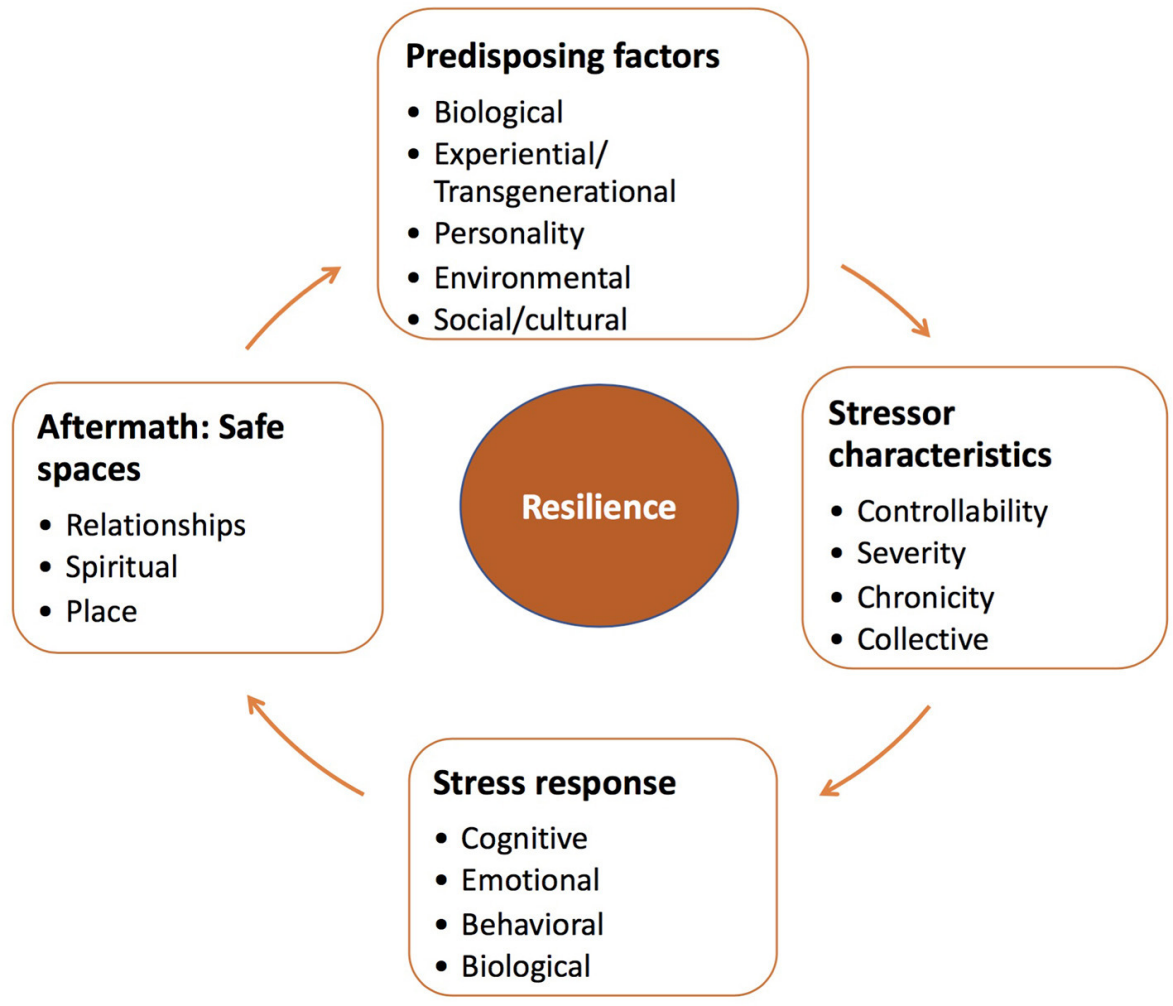
Resilience is regarded as a process that entails the dynamic and reciprocal relationships between features of the individual and their environment that influence (and are potentially triggered by) particular characteristics of the stressor and its appraisal. These appraisals may give rise to various responses to contend with the stressor (which might alter the stressor or bring about other stressors or protective factors). In the aftermath of stressor events, resilience might be fostered as a function of resources and processes that bring about feelings of safety and reassurance.

Figure 1 provides a broad overview of factors that contribute to resilience over the course of the stressor experience and in its aftermath. In this regard, we focus on several pre-disposing elements that shape responses to stressors. Such responses vary as function of both the objective characteristics and subjective appraisals of the stressor, which can interact with emotional, behavioral, and biological responses. Furthermore, following termination of a stressor, a series of adaptive changes can occur that might favor resilience and well-being, but failing these changes, vulnerability to pathology may be exacerbated. Finally, much like predisposing factors that shape stress processes during ongoing challenges, psychosocial resources may serve to re-establish safety and security that can contribute to sustained resilience to the impacts of previously experienced stressors, as well as those that might be encountered in the future.

Understandings of both vulnerability and resilience often adopt a linear approach to the phases of responding to a stressor, while at the same time acknowledging that such processes are multidimensional, synergistic, and reciprocal. We were tempted to review the literature in much the same manner. Instead, to better illustrate the interplay of features, we have opted to convey a few key elements that might contribute to resilience by framing them within the context of specific stressor scenarios. These scenarios are based on some areas of research in which we have been actively engaged to understand stress dynamics, namely (1) psychological abuse in an intimate relationship, (2) the experience of refugees to Canada, and (3) intergenerational trauma among Indigenous Peoples as a result of the Indian Residential School system. Through these scenarios, we provide fundamental information regarding central biopsychosocial aspects of resilience and vulnerability. In so doing, we emphasize the factors that contribute to the creation of safe spaces that enable individuals or groups to grow and thrive. In presenting each of these scenarios, we opted to consider a limited number of resilience factors relevant to them, but we underscore at this point that many of the components that contribute to resilience in any given scenario are also applicable to the other stressor situations.

## Resilience

Before considering the factors that favor resilience, we provide a broad overview of what resilience is often thought to entail, including several elements that have received less attention even though these may have fundamental repercussions to well-being. Resilience may reflect the propensity of an individual to overcome an illness, or it can refer to the ability of an individual to withstand the effects of stressful events that would ordinarily lead to pathology. Resilience may also entail a process of adjustment, transformation, and even growth (Ungar, 2008; Allen et al., 2014). Furthermore, although vulnerability and resilience are frequently considered to sit at opposite ends of a continuum, this is not fully accurate. An individual who is vulnerable to illness owing to the presence of certain genes or particular stressful experiences, might also be endowed with other genes or have had experiences that enable them to act against such risk factors. Likewise, the presence of a supportive social relationship, or having particular coping skills may allow individuals at risk to endure stressful events, thereby mitigating illness.

On the flip side, a person may be bestowed with numerous protective characteristics that favor resilience to diverse challenges, but there may be one simple feature that renders ineffective other protective factors in response to a given challenge. By example, the presence of a gene mutation that causes cancer development may override other factors that might ordinarily contribute to resilience. This said, to a considerable extent, understanding what makes individuals vulnerable to the negative impacts of stressors might also inform the processes that foster resilience. This includes individuals' subjective interpretations of events, their emotional reactions, the resources and skills they have available to cope with the event, and biological alterations that underpin vulnerability or resistance to illness. In effect, the search for features of the situation, or of the individual, that increase vulnerability to behavioral or physical disturbances revealed several factors that fostered resilience.

In response to common stressors encountered on a day-to-day basis, individuals' behavioral coping methods and stress-relevant biological systems ought to operate sufficiently well so that the risk of pathology emerging is low. As stressor severity increases, especially if it is uncontrollable, unpredictable, and experienced chronically or repeatedly, the load placed on biological systems increases commensurately. Among some individuals, the combination of effective protective behavioral and neurobiological processes may promote resilience, even in the face of multiple risk factors. In others, however, the continued strain may undermine behavioral coping methods so that greater pressure is placed on biological defense systems. Ultimately, a weak link in a component of such a system may give way, culminating in the emergence of a particular pathology. For one person this might comprise the provocation of diminished immune responses, and hence the emergence of related diseases. For another it may involve a cardiovascular disturbance that develops owing to chronic inflammation, or it might entail changes of neuroplasticity that promote psychological disorders, such as chronic anxiety, depressive disorders, or posttraumatic stress disorder (PTSD). Moreover, even when an individual appears to be resilient in the face of a stressor owing to a specific neurobiological process, this does not necessarily imply that this will hold in the long run. For example, short term inflammatory changes might be adaptive under certain conditions, but if these persist for extended periods, they may have multiple adverse actions, promoting depression, metabolic syndrome, type 2 diabetes, and heart disease (Mastorci et al., 2009; Anisman et al., 2018; Burgueño et al., 2020). By the same token, some behavioral coping strategies (e.g., avoidant coping) might be effective in the short term but could potentially undermine the adoption of strategies (e.g., obtaining help) that are needed to contend effectively with the stressor in the longer term.

With this said, it should be asked whether there are general features of individuals or particular experiences that favor resilience? For example, it has frequently been maintained that positive and nurturing early life experiences may act in this capacity by affecting neuronal plasticity or changes in gut microbiota (Kentner et al., 2019), and might even prime biological systems such that later challenges will be met by moderate neurochemical changes that enhance coping abilities (Anisman et al., 2018). At the same time, encountering stressful events that are experienced as tolerable could potentially facilitate learning how to appraise and cope with subsequently encountered challenges (McEwen, 2020). In effect, resilience is not a generalized state of the individual, but instead comprises multiple adaptations that might occur in response to challenges encountered within several domains and over time.

More commonly, resilience was attributed to a constellation of processes that reflect positive worldviews, such as altruism, social bonding, adaptive social behaviors, and appropriate responses to fear-related situations (Charney, 2004), perhaps owing to variations of oxytocin, operating in conjunction with dopamine variations within the nucleus accumbens (McQuaid et al., 2014). Relatedly, resilience might entail the ability to adhere to a positive perspective on life, eudaimonia (happiness or flourishing), accepting change, maintaining control, spirituality, as well as particular cognitive abilities, such as being adaptable and flexible in response to challenges (Belsky and Pluess, 2013; Gabrys et al., 2018). Certain personality dimensions may likewise contribute to resilience, including high levels of self-esteem, self-efficacy, mastery, optimism, extraversion, self-empowerment, hardiness, hope, and an internal locus of control (e.g., Carver and Connor-Smith, 2010; Jeste et al., 2015). Resilience has also been tied to having a strong social identity, being positively connected to others, and having an effective social support network (Haslam et al., 2014, 2016). In this regard, socio-ecological frameworks for understanding resilience have emphasized culture, spirituality, intergenerational relationships, and connections to the land (Kirmayer et al., 2011; Toombs et al., 2016). In essence, resilience emerges as a relational process involving the interplay of individual, social, cultural, and environmental factors (Liebenberg et al., 2015). Thus, beyond the outcomes ordinarily attributed to resilience, it has been maintained that it is as much a social process as it is an outcome (Norris et al., 2008).

Also entering the mix are multiple neurobiological elements, including numerous genetic factors that code for specific processes (e.g., neuropeptide Y or particular neurotrophins) and particular epigenetic changes may similarly imbue individuals with greater resilience (e.g., La Greca et al., 2013; Schmeltzer et al., 2016). These epigenetic changes could result in enhanced immune or endocrine functioning that are essential for adaptation (Mehta et al., 2020). Similarly, while some inflammatory processes have been associated with varied illnesses, epigenetic changes can act against cytokine-mediated inflammation and might thereby protect against stressor-related psychological and physical disturbances (e.g., Wang et al., 2018). Likewise, other stress-related hormonal factors (e.g., corticotropin releasing hormone, vasopressin, oxytocin, natriuretic hormones, angiotensin, neuregulins, some purinergic substances, and especially inflammatory factors) might be fundamental in accounting for the development of, or resilience related to comorbidities that are frequently apparent between stress-related psychological conditions and the appearance of metabolic syndrome, type 2 diabetes, and coronary artery disease (e.g., Zapata-Martín Del Campo et al., 2018).

## Stressor Characteristics and Appraisals

There are certain features of stressor events that may be more or less likely to create adverse outcomes. Ordinarily, severe stressors are more likely to favor pathology than are mild challenges. As a result, it might be thought that physical assault of one's intimate partner is more serious than psychological aggression. It might be expected that fleeing ethnic genocide would have greater pathological repercussions than encountering day-to-day discrimination in the country in which refuge was obtained. Or that the trauma and abuse experienced directly by survivors of the Indian Residential School system would be greater than the transgenerational trauma experienced by descendants. Yet in all of these instances, while the obviously severe traumas are unquestionably stressful, experiences with milder ongoing stressors were also found to be strongly linked to subsequent psychological disturbances (e.g., Matheson et al., 2007; Jorden et al., 2009; Bombay et al., 2014, 2019; Arriaga and Schkeryantz, 2015). In effect, to a considerable degree, the impact of stressors is determined by subjective appraisals of the experience, which may vary on the basis of the context in which they were experienced, as well as on the basis of earlier trauma encounters. As a result, consideration of objective features of a stressor is intricately connected to subjective appraisals.

In response to a potential stressor, individuals appraise whether the stimulus represents a threat. This is followed by a secondary appraisal concerning their belief that they have the capacity to deal with it effectively (Lazarus and Folkman, 1984). The appraisals that individuals make might be influenced by previous experiences in dealing with both similar and dissimilar challenges, or they may stem from specific beliefs, self-perceived abilities, as well as personality differences (Anisman and Matheson, 2005). When appraisals reflect threat of harm or loss, negative emotions such as anxiety or anger will ensue, whereas appraisals that frame the stimulus as a challenge or as an opportunity might elicit positive, motivating emotions.

Of particular relevance to this review is the importance of events that transpire after a stressful event has ended, and the implications for biological processes that underpin the onset of stress-related pathologies. Following a stressful experience, it is important that biological systems normalize relatively quickly. Aside from the unnecessary use of critical resources, a sustained neurobiological stress response may give rise to brain neuronal and peripheral processes operating excessively, which could lead to adverse outcomes (McEwen, 2007). In rodents, upon termination of an aversive event, stress-related neuronal activity may persist as long as the animal remains in the stress environment. Yet, this forced exposure may result in extinction of the stress response, including alterations of stress-related neurochemicals in rodents (Stockhorst and Antov, 2016), just as it does in humans treated through exposure therapy to diminish symptoms of anxiety related to specific cues, as in the case of phobias (Foa and McLean, 2016). It may be particularly significant from a resilience perspective that in an animal stress paradigm, normalization of the stress response can be promoted through the introduction of a safety signal (Minor et al., 1990), which might have its effects through actions at the insular cortex (Christianson et al., 2008) that subserves multiple functions, such as decision-making, risk prediction, and complex social behaviors (Gogolla, 2017). It appears that some brain regions operate in response to the here and now, whereas others also influence future responses to stressors.

Feelings of safety may be fundamental in turning off the stress response. Failure to achieve feelings of safety, possibly owing to dysfunction of neuronal circuits that permit feelings of danger to abate, has been associated with the development of anxiety-related disorders (Szechtman et al., 2020). When a stressor situation is one that does not permit feelings of safety to emerge, the activity of the anterior insula and amygdala are elevated (Tanovic et al., 2018). The elevated neuronal activity at these sites may reflect continued efforts by the brain to make sense of the situation. It has indeed been suggested that safety may be a learned response that fosters feelings of security and protection, but if safety is not established features of pathology (e.g., hypervigilance associated with PTSD) may evolve (Kong et al., 2014).

Similarly, it is fairly well established that having a negative ruminative coping style may be tied to later development of depressive disorders (Nolen-Hoeksema, 1998), and angry rumination has been associated with cardiovascular reactivity (Busch et al., 2017). In a sense, ruminating over a past event reflects an inability to ‘let go’ and in this way might have carry-over consequences that are not unlike those that accompany a persistent sense of being unsafe upon stressor termination. Negative rumination and worry represent the mind never coming to rest, and not having the opportunity to recuperate (Yan et al., 2013).

In the subsequent sections of this review, we will provide a series of scenarios that allow us to elucidate key appraisal processes that give rise to particular behavioral and biological coping responses. Such responses may directly influence stress-related outcomes. However, resilience to these outcomes, and indeed, psychological growth may ensue when individuals or members of a threatened group are able to cultivate cognitive, emotional, or physical safe spaces. Various strategies for achieving safety in the aftermath of differing stressor experiences will be considered.

## Scenario 1. Psychological Abuse in an Intimate Relationship

Psychological abuse frequently occurs within heterosexual dating relationships, as well as within the family home. While less visible than physical abuse, the effects of psychological abuse appear to be insidious with substantial impacts on the well-being of the victim. Although the perpetrator and target of such abuse cross gender lines, male partners are more commonly the aggressor, and women the target (Heise et al., 2019). Critical aspects of intimate partner abuse are that it represents a betrayal of the person who is supposed to be a trusted source of support and affirmation, undermines the victim's beliefs in her own worth and place in the world, and creates a home environment that is no longer a safe haven. Some women find the strength to terminate their abusive relationships, to transition from a life of being controlled to being in control, and to be resilient to the potential long-term impacts on stressor-induced outcomes, including depression, anxiety, substance use, or PTSD (Anderson et al., 2012).

### Stressor Controllability

Among those who have not experienced psychological abuse, there may be a tendency to underestimate the power of the persistent and repeated insults, passive aggression, and gaslighting on the victim's confidence, feelings of agency, and ability to take control of her situation. Psychological abuse is about the perpetrators' need for control and dominance, and the subjugation of their female partner (Ubillos-Landa et al., 2020). As described earlier, of the various features of a stressor situation that favor behavioral disturbances, control over the stressor has been most widely studied (Maier and Seligman, 2016; Anisman et al., 2018). Many of the features of psychological abuse inherently undermine women's appraisals of control, including its unpredictability, volatility, uncertainty (“is that really what he meant”), ambiguity (“did I do something to deserve it”), and complexity (“does he despise me or love me”).

Typically, uncontrollable events are more likely to promote behavioral disturbances than are controllable stressors. Some theorists attributed these variations to cognitive changes (e.g., learned helplessness), whereas others attributed these outcomes to particular stress-related neurochemical alterations (e.g., norepinephrine activity in the prefrontal cortex, dopamine in the nucleus accumbens) (Anisman et al., 2018). Relative to controllable stressors exposure to an identical uncontrollable stressor regimen in rodents resulted in greater turnover of norepinephrine and serotonin functioning across several brain regions, such as medial prefrontal cortex, hippocampus, amygdala, and hypothalamus, varying between strains of mice (Kasabov et al., 2019). The uncontrollable stressor regimen induced a reduction in the levels of norepinephrine and serotonin, possibly reflecting utilization of these amines exceeding their synthesis (Anisman et al., 2018). Furthermore, stressors promote brain region-specific variations of several neurotrophins, such as brain-derived neurotrophic factor (BDNF) that may be relevant to disorders such as depression (Duman and Li, 2012). It is of particular interest that although stressors generally reduce hippocampal BDNF, expression of BDNF was increased within the anterior cingulate, more so in response to a controllable rather than an uncontrollable stressor. The greater effects of the controllable stressor in this instance might reflect the adoption of active coping efforts, or to learning about the controllability of the stressful situation (Bland et al., 2007). In recent years, many of the learned helplessness advocates have come around to recognize the neurochemical underpinnings of the behavioral disturbances and have offered their own views regarding the specific neurobiological processes that contribute to behavioral disturbances (Maier, 2015). This has included variations of BDNF, as well as serotonergic changes. In this context, not only do controllable and uncontrollable stressors differentially influence serotonin functioning, but it also appeared that rodents that had encountered a controllable stressor did not exhibit the behavioral disturbances and serotonergic changes ordinarily associated with a subsequent uncontrollable stressor experience (Amat et al., 2010). In essence, learning that a stressful situation is controllable may immunize the organism against subsequent adverse effects that stem from a subsequent uncontrollable stressor.

Relatedly, from a resilience perspective, the physiological (and subsequent behavioral) impacts that occur in humans can be influenced by previous experiences with controllable stressors. If women who experience abuse have learned safety strategies for contending with mild interpersonal challenges in their current or previous relationships (e.g., possibly confronting the insults, or by turning to others who re-affirm her self-esteem), they might be able to manage or terminate the relationship if the abuse continues (Wood et al., 2019). Ordinarily, when a challenge first occurs, in the absence of previous relevant experiences, it may be unclear whether the stressor is controllable or uncontrollable, or whether it will be a brief insult or one that will be prolonged or repeated. Indeed, this is often the case with abuse, as it might start in small ways that can initially be ignored but evolves over time, as women increasingly question their own worth in response to repeated challenges. But in the first instance, some neurobiological changes ought to occur rapidly and relatively strongly, and then, once the characteristics of the stressor are understood, these responses could be down- or up-regulated as necessary to contend with it effectively. Thus, hormones, such as hypothalamic corticotrophin releasing hormone, pituitary adrenocorticotropic hormone (ACTH), and adrenal cortisol (corticosterone in rodents) ought to respond quickly, and hence, might initially be less influenced by stressor controllability, relative to several neurotransmitters (Anisman and Matheson, 2005). As well, as described earlier, within the anterior cingulate cortex, which is involved in the processing of emotionally salient events as well as in decision making, BDNF expression was elevated to a greater extent after controllable rather than uncontrollable stressors (Bland et al., 2007). This may reflect the possibility that BDNF was in some fashion related to new learning associated with a controllable situation rather than being a consequence of the stressor itself. Thus, it is tempting to suggest that learned controllability contributes to resilience, but at times such a response might be counterproductive as this view is predicated on the assumption that appraisals of controllability are actually accurate. As we know, humans may not be accurate in their appraisals and in decision making (Tversky and Kahneman, 1974). If controllable events are appraised as being uncontrollable, individuals may cease making efforts to determine their own destinies (as is too often the case among women in abusive relationships), they may seek exceedingly complex solutions to contend with these events, or they might adopt counter-productive strategies (e.g., substance use). Conversely, if uncontrollable events are appraised as controllable, then individuals may persist in fruitless efforts to alter the situation (e.g., repeatedly excusing or forgiving the abusive behavior; Ysseldyk et al., 2009).

### Behavioral Coping Strategies

Based on the appraisals (or misappraisals) that are made, specific coping strategies will be endorsed, although to an extent these coping methods may also reflect past experiences and dispositional propensities (i.e., coping styles). If individuals are able to enlist effective methods of coping, stressor-related problems ought to be relatively limited. In contrast, adopting poor coping methods that do not effectively manage the stressor or the emotions it elicits may favor the development of pathological conditions.

A wide range of coping strategies can be used to deal with stressors. These are often viewed as falling into three broad categories: problem-solving, emotional expression, and avoidance. Although specific coping behaviors are often assigned to one category or another, in fact, most coping methods do not fall exclusively within a single category. Instead, particular behaviors can serve in multiple capacities, varying with the nature of the stressor, the context in which it is encountered, and over the course of time (Anisman and Matheson, 2005). For example, a woman in an abusive situation might seek social support in order to obtain information that allows her to determine how to effectively manage or leave her situation, or she might seek support in order to avoid thinking about the abusive circumstances she experiences at home. Thus, although in both instances, women are seeking support, the purpose differs.

It should be underscored that particular coping behaviors are not typically used in isolation of other strategies. It may be that adeptness in appropriately using several coping strategies in conjunction with one another is most likely to promote resilience. For instance, rumination that is accompanied by self-blame, recrimination, and emotional expression, may favor the occurrence of depression (Nolen-Hoeksema, 1998), as well as PTSD (Spinhoven et al., 2015). Unfortunately, this pattern characterizes the coping profile often endorsed by women experiencing psychological abuse (Matheson et al., 2007). Conversely, rumination accompanied by problem-solving and strategies to manage emotions, as well as cognitive disengagement is not predictive of depression (Kelly et al., 2007). Being skilled at using a relatively broad range of coping strategies, and flexible in the choice of behaviors, so that the individual is able to shift from one strategy to another as the situation requires, may be the ideal approach to deal with stressors (Cheng et al., 2014; Juster et al., 2016). Being flexible in appraising stressors may similarly be advantageous (e.g., recognizing when a situation is controllable and when it is not), and may contribute to effective coping (Gabrys et al., 2018).

A common incorrect assumption is that emotionally expressive coping is a maladaptive strategy, given that it is often associated with depressive disorders. Yet, emotional expression may be instrumental in acknowledging, exploring, and understanding emotional responses to challenges, and may be fundamental in helping individuals come to terms with their feelings, consequently diminishing distress (Austenfeld and Stanton, 2004). Particularly when the stressor is uncontrollable, strategies that enable the individual to manage their emotional reactions might indeed be the best way to contend with the situation. In this regard, women's use of emotionally expressive coping strategies might signal a request for help from others in her support network. Conversely, emotionally avoidant coping among women in abusive situations can provide them with some degree of illusory control, at least in the moment (Matheson et al., 2007). In actuality, there is no single strategy that is best to deal with stressors across all situations, and instead coping methods ought to vary with the nature of the stressor and the context in which it is experienced.

### Relationships and the Social Safety Net

The extremely detrimental effects of experiencing abuse from an intimate partner (exacerbated by the isolation from others that is often imposed by abusive partners) is testament to the importance of social connections for well-being. Strong and supportive social relationships are a fundamental protective factor contributing to stressor recovery and resilience (Hobfoll et al., 2007; de Terte et al., 2014; Karmel et al., 2020), including in the aftermath of intimate partner abuse (Anderson et al., 2012). The nature of such relationships might vary in importance over the life span, shifting, for example, from familial caregivers (e.g., parents), to peers, to intimate partners and spouses, and to offspring (Wrzus et al., 2013). In this regard, it has been suggested that it is not simply the number of people within a support network that is important, but rather, the perceived availability of an appropriate source of support that is matched to the stressor at hand (Cutrona and Russell, 1990; Rini and Dunkel Schetter, 2010). Such matching refers to the support person's capacity to provide the type of support needed in response to a given situation, which might range from emotional reassurance (e.g., from a close friend) to having the skills or knowledge to help address the problem (e.g., a helping professional).

While perceptions of support availability are critical to providing the individual with the confidence and assurance that they can contend with a stressor, the actual receipt of support is less consistently associated with positive well-being outcomes (Uchino et al., 2011; Guilaran et al., 2018). Enacted support carries a complex set of implications, ranging from indebtedness, to its effects on perceived self-efficacy, to say nothing of its responsiveness to the problem at hand, and the erosion of support that can occur over time (Maisel and Gable, 2009; Ren et al., 2018; Ross et al., 2020). Such erosion is not an uncommon experience among women in abusive relationships. Sources of support become increasingly fatigued and frustrated by her inability to terminate the relationship, and less willing to provide support over time (Waldrop and Resick, 2004; Anderson et al., 2012). As a result, women in abusive relationships are less likely to reach out, and friends and family are uncertain how to respond when they do (Zautra, 2014).

In this regard, social support seeking can be a double-edged sword. When the individual turns to a person from whom they anticipated support, an unsupportive response can be highly distressing (Jorden et al., 2009; Woods et al., 2020). Such unsupportive interactions might exacerbate engagement in ineffective coping strategies (e.g., self-blame), and undermine perceptions of a supportive social safety net that had been counted upon (Manne et al., 2019). Much like appraisals and coping processes, for a social safety net to be effective, there needs to be flexibility within the support system. This serves both to ensure appropriate support for the specific stressor, as well the ability to identify an alternative source of support when a key resource falls through (as in an abusive relationship) (Laireiter et al., 1997).

Perhaps for this reason, and particularly given that humans are the ‘ultra-social animal’ (Tomasello, 2014), being imbedded within a relevant social group (Kaniasty and Norris, 2009; Uchino et al., 2018), or having a sense of belonging to multiple social groups affords benefits to well-being (Haslam et al., 2009). Multiple groups (e.g., sports teams, family, work colleagues, church members, home community) likely reflect different social functions, and as a result, the more groups that an individual identifies with, the more resilient they appear to be in the face of personal, social, and physical stressors (Iyer et al., 2009; Jetten et al., 2015). Even when an individual's group identity is threatened (e.g., through stigma, discrimination, or political violence), which ought to result in numerous negative psychological and physiological stress responses (Matheson and Anisman, 2012), highly identified individuals often experience the least distress (Cruwys et al., 2014). Perhaps emanating from evolutionary advantages (Tomasello, 2014), the groups to which a person belongs provide key social and tangible resources that enable individuals to accomplish goals, including social change through political action, which would otherwise be unattainable at the individual level (Haslam et al., 2009). In addition, belonging to multiple groups provides the flexibility needed to address various stressors, along with the capacity to turn to any one of multiple people within the support system (Haslam et al., 2016), or to create an identity segue to draw on new groups following a life transition (Ysseldyk et al., 2013). For all of these reasons, the multiple benefits of social identification and connectedness has been referred to as the “social cure” (Jetten et al., 2017).

Although the perceived availability of at least one trusted source of support appeared to be the most important factor associated with resilience among women leaving an abusive relationship, identifying such support after the prolonged restrictions associated with experiencing a controlling abusive partner might be a challenge. Despite the erosion of support women experienced while in the relationship, those who were resilient in the aftermath found that informal networks (e.g., family) were willing to re-engage once they had left the abusive partner (Anderson et al., 2012). Other sources of support exist within the community, including peer supports. Indeed, once women have re-established their lives, peer support and volunteering can be an effective strategy not only for building a social safety net, but can often promote the ability to find self-acceptance and meaning in the abusive experiences, which further contribute to long-term resilience and well-being (Zautra, 2014).

While social safety nets are most often considered in relation to human social supports, there is mounting evidence that supportive relationships can be in the form of companion pets or service animals. Just as many women in an abusive situation may feel unable to leave due to concerns about her children, they may also be unwilling to leave behind a beloved pet that might not be welcome in transitional housing (Barrett et al., 2018; Stevenson et al., 2018). Owning a pet is associated with positive benefits, such as helping the individual maintain a positive self-image, increasing quality of life, reducing anxiety, or hyperarousal associated with PTSD, or even providing a basis for strengthening human social connections through social participation (Wood et al., 2015; Brooks et al., 2018; Wells, 2019). Companion animals might be especially important sources of support and safety for those populations who are socially marginalized (Brooks et al., 2016, 2018), as animals are perceived as non-judgmental, providing unconditional acceptance that may be lacking in more traditional relationships (Brooks et al., 2016). For example, among homeless youth, having a companion pet helped mitigate the stressors associated with street life, reduced youths' engagement in risky behaviors (e.g., substance use), provided them with a safe and secure attachment figure, and gave youth the opportunity to experience the compassionate side of humanity (Lem, 2016). Homeless women in particular (many of whom have experienced previous abuse) recognized the therapeutic value of pets in providing them with companionship, unconditional acceptance, and a sense of personal safety (Labrecque and Walsh, 2011). War veterans experiencing PTSD symptoms also benefitted from service dogs trained to meet their psychological needs (Beetz et al., 2019), not only providing them with an ongoing sense of reassurance and safety, but in addition resulting in a decrease of PTSD symptoms over time (Kloep et al., 2017; O′Haire and Rodriguez, 2018), as well as co-occuring problematic substance use (Husband et al., 2020). Although there is still a need for evidence-based research to fully understand the effects of animal-assisted interventions (Fine et al., 2019), having a pet appears to provide the individual with two fundamental factors associated with resilience, namely a secure connection and greater feelings of safety (Zilcha-Mano et al., 2011).

## Scenario 2. the Refugee Experience

In the face of violent national ethnic or religious genocides, citizens flee their home countries to seek refuge in receptive nations. Many refugees have experienced the death of loved ones, been subjected to torture, witnessed the violent apprehension of family members, friends and neighbors, survived horrendous conditions in refugee marches and camps, and feared for their own and their family's survival. Once established in a host country, refugees must often learn a new language, a new set of cultural and social norms, find employment and housing, and sometimes continue to worry about family members who were unable to escape; those who belong to a racialized group may also encounter negative stereotypes and discrimination. In effect, the refugee experience is one of social and physical displacement and turmoil. While such severe and ongoing stressors are often associated with a range of stress-related pathologies, many refugees not only survive the experience, but flourish and create a new life for themselves and their families and make strong contributions to their new home communities.

Notably, just as for women in abusive relationships, refugees' appraisals of control over their situation may contribute to their resilience upon resettlement. The capacity to enact behaviors to establish themselves in a new country (e.g., learning a new language) might contribute to such perceptions of control. In contrast, other experiences, such encounters with continued discrimination could engender feelings of uncontrollability, and in addition challenge perceptions of the supports available to build a new life (Jorden et al., 2009). Perhaps for this reason, the creation of community social networks that decrease refugees' feelings of isolation and increase feelings of empowerment play an important role in their capacity to adapt to a new setting and to demonstrate resilience in the face of subsequent stressors (Block et al., 2018).

### Sensitization of Neurobiological Processes

Despite having physically left behind an unimaginably traumatic situation, memories and emotions associated with their past experiences continue to have a presence in the lives of refugees. An important feature of stressors is that their effects, even those that are experienced acutely, can have marked impacts that persist long after the initial experience. Although the majority of neurobiological changes in response to acute stressors are short-lasting, being apparent for a matter of minutes or hours, these neurochemical processes can be sensitized (primed) so that when animals are again exposed to the stressor at a later time, these changes will readily be reinstated (Anisman et al., 2003). Indeed, some of these actions can be engendered by cues that had been associated with the stressor (in human research these cues are described as “triggers”). These sensitizing actions might not be observable on a day-to-day basis but may be pronounced upon subsequent exposure to a stressor. Sensitizing actions are not necessarily limited to responses when the same stressor is encountered, as cross-sensitization can occur so that an exaggerated response is elicited when a different stressor is subsequently encountered. For example, studies in animals demonstrated an exaggerated response among previously stressed rodents upon exposure to a different stressor, and was further apparent in response to drugs that affect monoamine activity, such as amphetamine or cocaine, as well as morphine (Anisman et al., 2003; Bland et al., 2003; Uban et al., 2015). Likewise, stressful experiences can result in the sensitization of pro-inflammatory processes so that responses to later immune signaling molecules (cytokines) are exaggerated (Anisman et al., 2003). Sensitization to stressors was initially observed in relation to norepinephrine and dopamine activity, but these processes have since been documented in relation to multiple neurotransmitters, hormones, growth factors, and inflammatory processes that have been linked to psychological and physical illnesses (Anisman et al., 2018). What this means for refugees is that the experiences from which they fled might continue to impact their responses to subsequent unrelated stressors, affecting underlying biological processes, along with appraisals and coping strategies (Matheson et al., 2008).

Predictably, reminder cues associated with a traumatic stressor may instigate profound neurobiological effects in animals and in humans (Matheson and Anisman, 2012). Moreover, periodic presentation of stressor reminders over the weeks following an initial trauma may limit the dissipation of processes that would otherwise permit the organism to recover (Maier, 2001). This has obvious implications regarding vulnerability to stressor-related pathologies, but might be an important feature with respect to the building of resilience. For instance, what are the features of the stressor (or the stressor context) that allow for an adaptation to the impact of stressors under some conditions (or in some individuals), but promote sensitization of neurobiological processes in other instances? In this regard, women in abusive dating relationships were found to show marked increases in cortisol levels in response to reminder cues of abuse, whereas ethnoracial minorities exposed to reminders of traumatic discrimination did not (Matheson and Anisman, 2012). Under what conditions might previous experiences with controllable stressful events allow individuals to become immunized against the adverse effects of subsequent stressor experiences? Might having effective social support or positive nurturing early life experiences act in a similar capacity to promote resilience in the face of later stressor challenges?

It has been maintained that although severe stressors, particularly those experienced early in life (e.g., witnessing the assault or death of a parent), may have lasting negative consequences, encountering *tolerable* stressors (i.e., those that can be overcome by personal resources or with effective social supports) might engender resilience, as the individual learns the effective use of certain coping strategies, or to be flexible in their coping strategies as the situation demands (McEwen, 2020). This said, stressors that on the surface appear tolerable (e.g., racial micro-aggressions; psychological abuse) can, in fact, create persistent distress that culminates in pathology, particularly when such *apparently* mild stressors are encountered repeatedly. In this regard, the well-being of Somali refugees to Canada was more strongly associated with assimilation stressors encountered than with their exposure to the violence in Somalia (Jorden et al., 2009).

### Coping by Meaning Making

Although traumatic experiences frequently engender negative outcomes, this shouldn't be misunderstood as implying that traumatic stressors will necessarily have such repercussions. There are instances in which intense stressors enhance resilience (i.e., “what doesn't kill us might actually make us stronger”), perhaps being dependent upon when the stressor was encountered (e.g., during childhood or as an adult), and the protective resources that were at hand to contend with it (e.g., a caring and empathetic source of social support).

Severe trauma undermines fundamental beliefs about the world (Janoff-Bulman, 2010), and the ability to generate a sense of coherence and derive positive meaning from the event may be a fundamental coping strategy. Individuals who had experienced trauma, but found meaning through their hardships (benefit finding, posttraumatic growth), were less likely to succumb to psychological disorders (Davis et al., 2000; Albuquerque et al., 2017) and were better able to cope with further challenges (Hamama-Raz et al., 2019). More than 70 years ago, (Frankl, 1946) reflected that, even if we are unable to avoid suffering, we can choose how to cope with it, perhaps *finding meaning* in it, and then move forward with renewed purpose and hope.

Along these lines, collective interpretations of the violence refugees experienced contributed to their capacity to find meaning and to carry on effectively with their lives. For example, war-related violence was interpreted by Somali refugees as a political assault on tribal lineage. As a result, rather than dwelling on their personal suffering, these refugees appeared to establish social norms that guided their interpretation and response to events, and this shared understanding may have limited psychological distress (Zarowsky, 2000; Elsass, 2001).

Collective interpretations do not always promote resilience but might instead promote feelings of loss (Jorden et al., 2009). Individuals from war-torn communities that dwelled on the violence and destruction of the social structure demonstrated greater distress than those from communities that focused on notions of resistance (Abramowitz, 2005). In this regard, the collective interpretations of refugees who are in exile might comprise a sense of loss and dislocation. Re-establishing a sense of connection with other refugees might help to recreate a counter-narrative of strength and agency. Social support may play an especially poignant role in the well-being among refugees by maintaining a shared interpretation of their collective experiences, and by providing social resources to contend with acculturation stressors (Jaskinskaja-Lahti et al., 2006). Perhaps for this reason, those who became actively involved in helping other refugees were better able to make sense of their experiences, and experienced less distress associated with their losses and acculturation stressors (Puvimanasinghe et al., 2014). Such *altruism born of suffering* has been found to promote healing and growth among trauma survivors (Vollhardt, 2009). It is suggested that such altruistic behaviors allow survivors to transform the meaning of past suffering in a manner that allows for psychological growth and collective resilience (Staub and Vollhardt, 2008).

### Spiritual Connections: Finding Meaning and Purpose in an Unsafe World

Traumatic events often challenge core values and beliefs about safety, self-worth and the meaning of life (Janoff-Bulman, 2010). Accordingly, in the aftermath of such experiences, espousing particular spiritual or religious world views, practices, and ceremonies is a common response to life-changing events (Peres et al., 2007), including in response to refugee trauma (Bryant-Davis and Wong, 2013; Adedoyin et al., 2016). Spirituality is sometimes viewed as a personal experience and set of practices, whereas religion reflects a commitment to a collectively agreed upon set of beliefs and doctrines recognized by a sacred institution. In both instances, however, they reflect individuals' connection to a higher entity, and in so doing provide life purpose and meaning (Starnino, 2016), perceptions of control in an unpredictable world (Kay et al., 2008), and serve as a basis for self-enhancement (Sedikides and Gebauer, 2010).

Although there is little question that the factors that contribute to resiliency in the aftermath of trauma vary cross-culturally (Ungar, 2008), turning to some form of religious belief appears to be a common source of re-assurance and meaning. It has been estimated that 84% of people globally affiliate with a religious group (Pew Research Centre, 2017). After the September 11 attack on the World Trade Towers in the United States, 90% of Americans allegedly turned to religion or spirituality to cope (Schuster et al., 2001). Religion places control of traumatic events in the realm of a higher order, allowing followers to derive a sense of coherence in the experience, that what happened was destined to be, and that there is reason to have hope for the future (Peres et al., 2007), which together bolster resilience (Masten, 2015). It is also possible for individuals to foster a sense of partnership with a divine being, working together to problem solve (Wilt et al., 2019). The stability of spiritual and religious worldviews over time and place may also satisfy the need for belongingness and offer confidence in the midst of uncertainty (Ysseldyk et al., 2010).

Religiosity also serves as an important social identity, and as a result may contribute to providing an effective source of social support. However, it is also unique in that it invokes guiding epistemological and ontological beliefs about human existence that are shared among group members (Ysseldyk et al., 2010). Historical and cultural continuity grounds these core beliefs in rites, symbols, and physical spaces created over millennia but adapted to fit with shifting social norms and ways of living in a given era (Haslam et al., 2010). Thus, religious affiliation is not simply another social identity, as its positive effects depend on the confluence of spiritual belief, social participation, and establishing a strong congregational social network (Lim and Putnam, 2010). In effect, individuals not only gain a sense of belonging, but also benefit from a set of guiding beliefs that offers a worldview entailing life purpose and meaning (Bryant-Davis and Wong, 2013).

This said, spirituality is not always an effective protective factor. It also happens that trauma can undermine beliefs in a higher power, as victims feel that “God has abandoned me” (Pargament et al., 2001; Bryant-Davis and Wong, 2013). This might be especially the case when individuals encounter a morally injurious event, which has been defined in numerous ways, but essentially involves events that damage a person's conscience or moral compass as a result of perpetrating, or failing to prevent, acts that transgress deeply held moral beliefs (Litz et al., 2009). The resultant moral injury has been referred to as a “a deep soul wound that pierces a person's identity, sense of morality, and relationship to society” (Silver, 2011). Although primarily studied among military personnel exposed to combat, such events might also affect others exposed to violence, such as refugees, journalists, front-line personnel in a natural disaster, or among hospital staff forced to weigh provision of treatment (Barnes et al., 2019). Such events can give rise to existential issues, wherein individuals question their moral worth, goodness in the world, and spiritual faith. While moral injury might co-occur with PTSD, they are mechanistically different, in that the latter is more likely to be fear-based (Barnes et al., 2019), and functional brain connectivity changes associated with moral injury and PTSD could be distinguished from one another (Sun et al., 2019). Moral injury (the potential outcome of encountering such an event) also differs from the consequences of other life-threatening traumas, being more strongly associated with post-event emotions [shame, guilt, (self-)contempt, fear of judgement], rather than those experienced during the event (Barnes et al., 2019). It has been suggested that healing moral injury might entail re-establishing interpersonal trust (through compassion and loving kindness) (Hinton et al., 2013), and resolving spiritual struggles (through forgiveness and repentance) (Pearce et al., 2018).

## Scenario 3. Indigenous Peoples' Collective and Historical Trauma

Indigenous Peoples in many regions of the world have been subjected to colonialist acts intended to eradicate their culture and traditions, if not the people themselves. In North America, European colonizers forcibly drove Indigenous Peoples off of their lands to the confines of reserves. As well, their children were abducted and placed in residential or boarding schools with the explicit goal of severing familial ties and cultural socializations while instilling Euro-Christian values, including an understanding of racial hierarchy. Concurrently, legislative policies were enacted that disempowered Indigenous Peoples, outlawed cultural and spiritual practices, and effectively rendered them wards of the state. As a result of historical trauma and ongoing systemic racism, current generations of Indigenous Peoples disproportionately experience high rates of suicide, substance use, depression, anxiety, diabetes, and respiratory illnesses, to name but a few (Gone et al., 2019; Menzies, 2019). At the same time, there are many strong Indigenous Peoples who are fighting to restore their cultural traditions, reclaim possession and self-determination of their lands, and overturn the racialized injustices and legislative acts of the colonizers.

It should be clear that the appraisal processes (controllability) and sensitization effects associated with early life and traumatic stressors are relevant to the experiences of Indigenous peoples, and as will be discussed, have repercussions across generations. Social relationships are core to Indigenous values and meaning-making, reflected in connections to family and community, and extending across generations from offspring to ancestors in the spiritual world (Kirmayer et al., 2012). Indeed, the holistic understanding of wellness that is fundamental to most Indigenous worldviews emphasizes the importance of relationships and the value of connection to all that is animate (Kimmerer, 2013).

### Stressor Chronicity and Allostatic Overload

Inherent to the concept of historical and transgenerational trauma is exposure to pervasive, repeated, chronic stressors that affect a group as whole. Although individuals may encounter numerous acute stressful events, those that appear most damaging to psychological and physical health are chronic challenges. Chronic stressors have been tied to severe psychological disturbances (e.g., depression, anxiety, PTSD, substance use), as well as a host of inflammatory-related physical disorders, such as diabetes and heart disease (Furman et al., 2019). The development of these disorders varies with several other variables, including previous stressful experiences, such as early life adversities (e.g., neglect, abuse, illness-related hospitalization, maternal depression, parental substance use), and was more prominent in vulnerable populations (e.g., those with subclinical illness features, in older individuals, and those living in poverty or who maintained poor lifestyles). Early life challenges stemming from transgenerational trauma associated with parental attendance at Indian Residential School are further related to a proliferation of stressors in childhood and adult life (Bombay et al., 2014, 2017), and the combined effects of the accumulation of multiple stressors may operate much like stressor chronicity (Steptoe and Kivimäki, 2013). It should be said that while early life stress emanating from abuse, death of a loved one, or domestic violence were linked with later depression, other stressors, such as illness and natural disasters, and even poverty were less likely to be related in a similar manner (LeMoult et al., 2020). These latter stressors may have been more responsive to protective social factors, including familial or community solidarity, as well as spiritual connections.

As discussed earlier, stressors ordinarily elicit multiple neurotransmitter, neuroendocrine, immune, inflammatory, and peripheral nervous system changes that are presumed to have adaptive value. These neurobiological adaptations (allostasis) operate together with behavioral, cognitive, and social processes to maintain well-being. As much as this is highly adaptive, should these neurobiological processes be engaged for protracted periods, *allostatic overload* may be experienced, which could provoke negative repercussions on particular brain neural circuits and body systems (McEwen, 2000). By example, persistent release of glucocorticoids could disturb hippocampal corticoid receptors, so that an essential shut-down mechanism for hypothalamic-pituitary-adrenal activation is impaired, resulting in sustained adrenal cortisol release, leading to further hippocampal disturbances (McEwen, 2007). Likewise, the changes of norepinephrine and serotonin utilization initially provoked by an uncontrollable stressor are elevated with a chronic stressor that might be met with a compensatory increase of synthesis. However, this presumably adaptive change can persist for only so long before maladaptive behavioral consequences are apparent (Anisman et al., 2018). The reduced hippocampal BDNF gene expression and protein levels induced by acute stressors (Duman and Monteggia, 2006) were still more pronounced following a chronic stressor regimen in rodents (Grønli et al., 2006). It appeared that the actions of a chronic stressor regimen on hippocampal BDNF were more pronounced in females than in males, which is in keeping with the greater depression vulnerability in human females (Liu et al., 2019). In addition to the differential effects of acute and chronic stressors on neurobiological processes, very different effects on immune functioning and the production of chronically elevated inflammatory processes have also been documented (Dhabhar, 2014).

Many different biological checks and counterchecks occur in an effort to maintain allostasis. For instance, the decline of monoamine levels provoked when acute uncontrollable stressors cause utilization to exceed synthesis might not be evident in response to chronic stressors. As a challenge continues, a compensatory increase of neurotransmitter synthesis may occur, meeting the high rate of utilization, so that monoamine levels may normalize. This may be highly adaptive in the short and medium term as it allows the organism to deal with ongoing challenges. It is tempting to assume that under these conditions the storm has been weathered and that things are under control. In fact, however, when increased utilization and receptor activation persist for an extended period, an excessive load is placed on critical systems. Likewise, chronic stressors may diminish innate and acquired immune functioning, and dysregulate inflammatory processes that favor the emergence or exacerbation of various illnesses if they already exist or are at subclinical levels (Anisman et al., 2018).

Allostatic overload is most apt to occur when the chronic stressor does not readily allow for adaptation (e.g., when the stressor occurs on an intermittent, uncontrollable, and unpredictable basis leading to still greater neuronal activity). In addition, individuals who experienced earlier or concurrent social and environmental insults, such as those related to work, home-life, poverty, and social factors may be especially vulnerable to the development of allostatic overload (McEwen and Akil, 2020). Given that diet, obesity, exercise, and sleep also affect many stress-relevant processes, including inflammation and microbiota that affect immunity (Cryan et al., 2019), these should also be included in the mix of factors that could affect allostasis and allostatic overload. Importantly, most stress-related illnesses do not simply appear overnight but reflect the accumulation of chronic small changes that develop with repeated insults over an extended period (Dube et al., 2003), pointing to the importance of evaluating the impact of chronic challenges in relation to disease occurrence.

These persistent effects of stressors can be detected throughout life irrespective of when the initial negative experience was encountered. Significantly, the proactive effects of stressors in relation to neurotrophins, inflammatory mechanisms, and gut microbiota were particularly prominent if they had been experienced during early life (Burns et al., 2018; Audet, 2019). Such outcomes may reflect the sensitization of neurochemical systems, or the introduction of epigenetic changes that manifested as altered responses to later challenges (Szyf, 2011; Torres-Berrío et al., 2019). Further to this, stressors over the lifetime can have cumulative actions that favor the development of pathological outcomes, more so if the actions of stressors had been “embedded” during childhood through epigenetic changes and then exacerbated by further stressor encounters (Szyf, 2019). The initial stressor experiences may provoke cascading neurobiological changes, essentially modifying the organism's developmental trajectory so that basal biological functioning was permanently altered. Regardless of the mechanism, it is certain that adverse early experiences can have long-lasting ramifications that can emerge at later times, particularly if a stressor was again experienced. Owing to the plasticity of brain networks, both positive and adverse early experiences are the seeds that are laid down to bloom at later times.

Quite literally, historical or transgenerational trauma presents challenges that occur over generations. Historical trauma refers to the suffering that results from a collective history of colonization and its implications for disrupting traditional ways of life, culture, and identity (Brave Heart and DeBruyn, 1998). This underscores the explicit connection between the trauma experienced by previous generations on the well-being of descendants, and the continued gaps not only in individual wellness outcomes (Gone et al., 2019), but the implications for ongoing collective rights, functioning, and systemic discrimination (Bombay et al., 2017). Moreover, the view has been entertained that the biological consequences of traumatic experiences can be carried across generations though epigenetic changes, so that the descendants of survivors may be more vulnerable to later stressor challenges (Szyf, 2015; Cavalli and Heard, 2019). At the same time, it is possible that epigenetic changes (and psychosocial processes) associated with historical trauma could instill individuals with greater adaptability and resilience (Lehrner and Yehuda, 2018; Yehuda et al., 2018). Such a possibility can be derived from studies in rodents indicating that prenatal stressors could promote epigenetic changes that were advantageous in the context of threatening situations (St.-Cyr and McGowan, 2015). In effect, although prenatal and early life stressors could produce disadvantageous epigenetic changes, their ultimate actions may be context dependent.

There has been impressive evidence from animal studies showing that epigenetic effects can be promoted by stressful experiences as well as by a variety of toxicants. For instance, epigenetic changes related to prenatal or early life stressful events have been observed in relation to genes coding for glucocorticoid receptors (Grundwald and Brunton, 2015; Turecki and Meaney, 2016), the serotonin transporter (Kinnally et al., 2010), estrogen receptor alpha (Champagne, 2008), inflammatory factors stemming from infection (Weber-Stadlbauer, 2017), as well as many other biological factors relevant to physical and mental health disturbances. These epigenetic changes can occur within germline cells (sperm or egg), and thus, could be recapitulated across multiple generations (Franklin et al., 2010; Bohacek and Mansuy, 2015; Jawaid et al., 2018). These transgenerational outcomes (i.e., those that span at least three generations) may well be responsible for diseases brought about by stressors, infection, and by pesticides (Franklin et al., 2010; Manikkam et al., 2014; Weber-Stadlbauer, 2017).

The data concerning transgenerational effects in humans are sparce given the difficulties of obtaining DNA from descendants of traumatized individuals. Nonetheless, as historically traumatized groups, including Indigenous Peoples in Canada, Australia, and the United States, have experienced marked health disparities (Bombay et al., 2014), it has been suggested that this might stem, in part, from epigenetic changes (Conching and Thayer, 2019; Phillips-Beck et al., 2019). To be sure, numerous factors that emanated from historical trauma could account for these outcomes (e.g., poverty, poor maternal care of infants, limited childhood education, lack of medical service, a sub-optimal intrauterine environment, continued systemic discrimination) making it exceedingly difficult to identify the relative contributions of these numerous variables, including the role of epigenetic influences (Gone and Kirmayer, 2020). As often said, however, “the absence of evidence, does not imply evidence of absence.”

A relatively small number of studies were reported concerning intergenerational epigenetic effects in humans. Among the offspring of holocaust (Shoah) survivors, the risk for PTSD (or subthreshold features) was elevated in a subset of individuals. This risk was most evident in relation to several parental and child characteristics (e.g., parental mental health problems, attachment quality, having both parents be Holocaust survivors), and having encountered their own stressors (Dashorst et al., 2019). Cortisol levels were reduced among children of Holocaust survivors, which has been observed in relation to PTSD under other circumstances. Interestingly, cortisol levels in offspring were inversely related to parental PTSD symptoms (Yehuda and Bierer, 2008). More to the point of this discussion, PTSD and depression among Holocaust survivors were associated with the increased presence of epigenetic changes within the FKBP5 gene (Yehuda et al., 2016) that has been associated with glucocorticoid functioning and systemic inflammation (Zannas et al., 2019). An epigenetic change of the FKBP5 gene was also apparent in their offspring; however, in this case epigenetic marks were reduced (Yehuda et al., 2016; Bierer et al., 2020). It is uncertain whether this epigenetic effect in offspring reflects a vulnerability factor for later pathology or an adaptive change to limit excessive cortisol output.

Like the studies conducted with the offspring of Holocaust survivors, severe prenatal stressors stemming from war-related trauma may also instigate epigenetic changes related to BDNF as well as cortisol-related processes (Monk et al., 2016). Aside from the influence of these severe stressors, maternal adversity in the form of nutrition scarcity, preeclampsia, smoking, and diabetes, were accompanied by epigenetic changes of glucocorticoid-related genes (Nagarajan et al., 2016). Not unexpectedly, among women who experienced interpersonal or community violence while pregnant, psychiatric illnesses and epigenetic changes related to glucocorticoid functioning were frequently observed in these women, their offspring, and in their grandchildren (Serpeloni et al., 2017). Curiously, however, if the offspring of women who experienced violence encountered subsequent stressors, then these psychiatric outcomes were not apparent. Among the offspring of abused women, epigenetic changes were apparent at genes that encoded the glucocorticoid receptor (NR3C1), and its repressor (FKBP5), which might allow for greater ability to limit stressor-provoked cortisol responses (Serpeloni et al., 2019). Paralleling the effects of interpersonal violence, among the children of prenatally stressed middle-eastern refugees, similar outcomes were observed upon later war-related stressor exposure (Serpeloni et al., 2019). It should be added that epigenetic actions related to glucocorticoid functioning is only one of many neurochemical, neurotrophic, and hormonal factors that have been linked to trauma experiences, and it remains to be established whether any of these are subject to transgenerational effects (Kertes et al., 2017; Simons et al., 2017).

Although research dealing with epigenetic changes, as well as with allostatic overload has often focused on the impact of chronic strain within individuals, the insidious threats that comprise sustained social disturbances, social conflict, racism, and poverty can engender *type 2* allostatic overload, reflecting systemic rather than individual stressors (McEwen and Wingfield, 2003). While this form of allostatic overload can seriously undermine well-being, its resolution is less individual and more institutional and political, requiring altered social structures to prevent the development of pathological conditions (McEwen and Akil, 2020). As much as individuals might believe that behavioral change that acts against stressors and promotes good health is related to their own self-efficacy, lasting impacts may be profoundly influenced by their social groups, community initiatives, and broad government policies.

### A Place Called Home: Connection to Land

While colonization dominates the historical context of Indigenous Peoples' current wellness, strength, and resilience can be derived through a connection to the land and places that ground people in an identity, self-affirmation, belonging, and safety (Hopkins and Dixon, 2006). Among Indigenous Peoples, such connections are further imbued with spirituality, and promote a continuity of relationships across generations. In this regard, spiritual connections are not limited to moral or existential beliefs, but might also have links within the tangible world, including spaces that provide us with a sense of being “at home” with ourselves or, much like religious beliefs provide individuals with a sense of belonging in the universe. Physical places can be both natural or built spaces (such as one's home, church or workplace). Such spaces might be broadly defined (e.g., “by the ocean”) or might entail very specific locations that are “grounded in local ecologies, through cultural knowledge and practices” (Gone and Kirmayer, 2020). In this regard, a *sense of place* refers to how individuals characterize, experience, use, and understand places, as well as functional, subjective, and emotional attachments to these places (Graham et al., 2009; Lewicka, 2011). Most places are shared spaces and therefore also provide the foundation for social connectedness, identity, and a sense of belonging (Qazimi, 2014). A strong social community associated with a place can serve as a protective factor against the negative impacts of other environmental stressors (e.g., poor living conditions) on well-being (Fong et al., 2019). When place-based social relationships become inaccessible (e.g., due to retirement, or migration), well-being is diminished (Atkinson et al., 2016). Conversely, during place transition, maintaining place-based social ties can alleviate feelings of dislocation and thus, act as a source of resilience (Chow and Healey, 2008; Scopelliti and Tiberio, 2010).

The relative importance and meaning of a place can vary based on cultural background, residential status, and personal history (Wilson, 2003). In particular, at the heart of many Indigenous cultures, the land is infused with meaning that is expressed through language, stories, music, and art. Resilience derived from the symbiotic relationship to the land often involves the integration of cultural identity with the features of specific places. This relationship not only reflects an appreciation of the physical features of a geographical location, but also encompasses non-physical elements, such as the social, emotional, and spiritual aspects that provide the foundation of identity, and a sense of community and belonging (Wilson, 2003; Graham et al., 2009; Qazimi, 2014). In this regard, Indigenous elders reported that a strong attachment to the land maintained through ceremonial practices, community involvement, traditional food sharing, and use of traditional language were essential to affirming land-based and cultural identities, which in turn, protected them from adverse health effects and promoted physical, mental, and spiritual healing (Tobias and Richmond, 2014). Reflecting beliefs in the healing properties of connecting to the land, programs that encourage land-based activities have been emerging across Indigenous communities as a strategy for building youth resilience (Walsh et al., 2018). The objective of these programs is to build confidence, esteem, cultural connections and positive relationships among youth that may help them to contend with the challenges they encounter (Hackett et al., 2016; Walsh et al., 2018).

Predictably, disturbances to a place that are beyond an individual's control can result in feelings of *placelessness* (Wilson, 2003). Such disturbances may be especially marked during exile or forced transitions (as in the case of refugees), such as the environmental dispossession forced on many Indigenous populations (Tobias and Richmond, 2014). Such dispossession can be direct as a result of major changes to landscapes (e.g., construction of railroads, highways, dams), pollution, and limited access to the land preventing its use for ceremonies and traditional practices. However, indirect dispossession can also occur when, for example, language is lost, as Indigenous languages are often grounded in meanings that have evolved in relation to the home land. In this regard, historical colonialist actions involving involuntary relocations and restriction of movement, together with ongoing assimilationist policies associated with education, loss of language, health and governance, have all contributed to the erosion of the knowledge of the land that was passed down through generations, including a holistic understanding of social purpose and meaning (Allen et al., 2014). At the same time, many Indigenous Peoples, even those living in urban settings, report strong identification with their home communities (reserves), despite the impoverished infrastructure (water, housing, community centers, schools) emanating from systemic underfunding. Such community (or neighborhood) identification is associated with positive mental health that overrides the lack of community resources and socioeconomic disadvantage (Fong et al., 2019), affirming the importance of place identity as a resilience factor.

Although connection to the land is an integral part of most Indigenous cultures, recognition of the healing properties of spending time in nature is also emerging in more mainstream interventions to promote resilience and wellness (Pálsdóttir et al., 2018). Several reviews have concluded that immersive nature experiences are associated with positive well-being, cognitive, and social functioning, and a reduction of stress and other risk factors associated with diminished mental health (Bratman et al., 2019; Mygind et al., 2019). Moreover, connection to nature was associated with self-reported personal growth (Pritchard et al., 2020). Nature experiences can range from spending time in urban green spaces, gardening, activities or outings in natural settings, and fishing, hunting or being in the wilderness; at the same time, there is little clarity on the kinds of experiences that contribute most to well-being, how much time in nature is needed to feel a sense of connection, or for whom such contact or connection is most beneficial (Frumkin et al., 2017). Numerous key qualities might contribute to the restorative effects of nature, including the provision of sanctuary or refuge, prospect (i.e., open space with vistas), and a safe and secure space (Pálsdóttir et al., 2018). Such features may serve to reduce stress and relieve mental fatigue, increase physical activity and social connections, expose people to improved air quality, and enhance immune function (Frumkin et al., 2017). In addition, access to such spaces might provide a *third-place* (outside of home and work) that might operate as a safe haven that facilitates well-being (Fong et al., 2020), which may be especially relevant to women seeking refuge from an abusive home environment. Although some of the benefits of third-places cater to social needs (Fong et al., 2020), they might also serve as a refuge that allows the individual to disconnect from day-to-day stressors and reconnect with nature.

While connection to the land might be a source of resilience, changes to the land as a result of climate change are not only dispossessing those who inhabit and rely on it, but is serving as a traumatic stressor in itself (Cunsolo-Willox et al., 2012). When the land is the main source of sustenance, the inability to predict environmental phenomena, or the lack of tools to contend with such changes, can have particularly devastating effects on well-being at both the individual and community level. In addition, when extreme events (e.g., fires, floods, hurricanes, earthquakes) force displacement, temporarily or permanently, there is the additional diminishment of the safety and security of displaced populations. Under these conditions, resources (e.g., financial, along with basic needs such as water, food, clean air) are stretched or no longer accessible, family, and community members may be divided or lost, safety, and security are undermined, and exposure to potential toxicants and disease are heightened. Thus, having once had a protective factor to which individuals could turn in the face of stressors, cumulative or extreme environmental changes may be distressing, particularly for those populations that were most vulnerable prior to such events (Johnson and Galea, 2011). Nonetheless, resilience in the aftermath of such challenges does occur. It has been suggested that resilience is fostered when the survivors of natural events are able to express and anticipate collective solidarity and cohesion, and to act cooperatively to draw on collective social support resources (Drury et al., 2019). The emergent properties of a “ground-up” shared identity may be fundamental to establishing the social behaviors that promote collective resilience.

## Discussion

The processes underlying resilience are complex, involving the dynamic and reciprocal interaction of many moving parts. We often consider the contributing factors in a causal flow, and indeed, this has allowed us to learn much about the mechanisms triggered by stressful events. However, this approach also has limitations. In this regard, we sought to depict several pathways that might be evident in relation to specific stressor contexts, and in particular considered how various underlying biopsychosocial processes and resources might contribute to longer-term resilience and well-being in the aftermath of traumatic stressors. These stressor contexts included women's recovery upon termination of an abusive intimate relationship, the adaptation of refugees who had fled violence to establish a new life in a host country, and Indigenous Peoples' reclamation of traditional sources of strength following historical and transgenerational trauma within their own nation. Our objective in doing so was to highlight the complex interplay among elements as they unfold over time and the course of the stressor. This said, although we incorporated particular processes within a given stressor context, these elements could have been equally applied to the other contexts. Specifically, each of these traumatic experiences may be subject to the sensitization of neurobiological systems; in each case, the distress is typically chronic, often with uncontrollable features leading to allostatic overload and the emergence of subsequent pathology. In addition, processes that promote the sense of safety that are fundamental to sustained resilience apply to all three contexts. In this regard, spirituality may be a key source of resilience among women in the aftermath of abusive relationships (Anderson et al., 2012), and is also a fundamental aspect of Indigenous wellness (Toombs et al., 2016). Just as connecting to a place called home is central to the wellness of Indigenous Peoples, the importance of rebuilding a safe place is clearly integral to feelings of safety and reassurance needed by women leaving an abusive relationship, and to refugees fleeing violence. Finally, social relationships, whether interpersonal, familial, or community-based contribute to the well-being of refugees (Jorden et al., 2009), as well as to Indigenous Peoples (Toombs et al., 2016). Thus, although we have contextualized elements that contribute to resilience to highlight possible pathways and turning points, these processes are relevant across many contexts, interacting with the features of each.

It might also be noted that our review primarily focused on responses to social stressors. However, there are many types of stressors that call upon similar mechanisms and elicit the same challenges in their aftermath. These might include responses to diagnoses of terminal illnesses, chronic pain, job loss or financial distress, and so on. While the nature of these stressors differs substantially from those involving social phenomena, they nonetheless elicit common stress processes, including cognitive appraisals, emotional reactions, and behavioral and biological responses. Although the response pathways to social and non-social stressors might differ, in the end, they likely rely on many of the same resources to promote resilience. For example, contending with a diagnosis of a terminal illness might instigate a range of shifting appraisal and coping methods as the individual comes to terms with the diagnosis and their own mortality (Antoni and Dhabhar, 2019). However, much like social stressors, there is little question that strong social supports, a spiritual (not necessarily religious) framework for understanding their place in the world, and finding safe emotional, if not physical spaces, that provide them with some respite, might all contribute to the psychological wellness.

This said, it is difficult to identify the specific variables that are most important in accounting for the ability to withstand diverse challenges, especially as each type of challenge may call for different resilience factors, vary across situations, and over the life span. Moreover, like the individual differences that exist in response to stressors, what constitutes resilience in one culture could be very different in a second culture, and could differ yet again in groups that have experienced cumulative, historical trauma, such as Indigenous Peoples in many countries who experienced multiple indignities over the years following colonization (Gone and Kirmayer, 2020; Matheson et al., 2021). Appreciating and understanding these cultural experiences may contribute to the development of effective interventions among at-risk populations (Ungar, 2008; Dell et al., 2011; Kirmayer et al., 2012).

Not only might various risk and protective factors act additively or interactively to contribute to resilience, but an individual may be resilient to some types of challenges and not to others (e.g., social strife vs. chronic pain may involve different processes). Some people may be more likely to develop particular stress-related outcomes, but not others (e.g., development of diabetes or heart disease, but not an autoimmune disorder). And sometimes the exertion needed to respond to one stressor might diminish the capacity to respond to others (Juster et al., 2016).

Both vulnerability and resilience might also develop through particular experiences, which may interact with genetic factors. For instance, adverse early life events are thought to have long term ramifications so risk for adult psychological (e.g., depression) and physical disturbances (e.g., heart disease) is elevated (Hodes and Epperson, 2019). The impact of these interactions varies with the age at which the adverse event was experienced, and as a function of gender and ethnoracial group membership (Tomfohr et al., 2016; Brivio et al., 2020). Behavioral disturbances are further linked to epigenetic changes, genetic factors or the presence of particular polymorphisms. For instance, in the presence of a specific oxytocin polymorphism, stressor-related negative outcomes were less likely to develop. It had been maintained that, in addition to its apparent role in social behaviors, oxytocin may influence the salience of experiences so that under typical conditions positive early life encounters have beneficial long-term consequences, whereas negative events have adverse effects. However, individuals with particular oxytocin-related polymorphisms might not gain from positive encounters, but at the same time they might also be less affected by negative early life events (McQuaid et al., 2014).

One of the challenges of research conducted to understand stress vulnerability and resilience is that, although we might organize and conceptualize variables in a causal manner, in fact the vast majority of research is correlational, and often relies on self-report and retrospective recall. Causal understandings, and in particular those associated with epigenetic, genetic, or brain neurochemical changes are largely based on studies conducted in rodents. As much as these findings are informative, there are limits to what can be achieved through these studies. We previously described the necessary conditions for an animal model to be a valid facsimile of human disorders, as well as some of the roadblocks that can limit their usefulness (Anisman and Matheson, 2005). Aside from the difficulties of translating from animal to human conditions, particularly in view of the more complex ways in which humans appraise, interpret, and cope with stressors, the fact is that stressor-related outcomes in animals may not be fully recapitulated in humans. The ways animals contend with stressors are far more restricted than the diverse array of methods used by humans, and key social and cultural processes that promote feelings of safety are uniquely human (e.g., spirituality), although social factors and safe places may also contribute to the well-being of animals. To be sure, studies in animals allow for analyses of the interactive effects related to genetic and stressor characteristics (Prakash et al., 2006), and even permit analyses of the role of various social features (Audet et al., 2014). However, they are constrained by the validity of the models in light of the vast array of the institutional and sociopolitical factors in which human stressors are imbedded (McEwen and Wingfield, 2003; McEwen and Akil, 2020). Finally, for ethical reasons, researchers cannot subject laboratory animals to the horrid and debilitating psychological and physical stressors that humans too frequently endure (often perpetrated by other humans).

We have suggested several factors that might contribute to feelings of safety in the aftermath of a traumatic stressor that ought to contribute to resilience against negative short and long-term outcomes. However, each of these pathways to realize feelings of safety and security carry risks that need to be considered. For example, while individuals might be encouraged to seek social support, the reactions of others are not always predictable or helpful. Likewise, nurturing one's spiritual beliefs provides a world view that is comforting, but at the same time events can occur that are so injurious that they might irreparably sever the safety and reassurance found in these beliefs, even potentially exacerbating self-blame and the view that one deserves the bad things that happened. Even safe places might be violated if they were the location of traumatic experiences (such as domestic abuse, civil war, natural disasters), leaving individuals with greater loss and sense of placelessness.

To protect against the fact that the world is in actuality not a safe place, as noted at the outset of this review, it will be important to identify the constellation of factors (signatures) that might contribute to individual flexibility to garner resilience. Doing so may comprise the amalgamation of the meaning of past experiences, including both strengths and vulnerabilities, with hopes for the future; one eye looking backward, and one looking forward (Shariff, 2020). For example, among Indigenous Peoples, wellness is derived from both a strong spiritual connection to ancestors and a dedication to the futures of their children and youth. Similarly, among groups that have experienced collective trauma, learning from the perseverance of survivors is a basis for subsequent generations to recognize their individual and collective strengths. The same connections between past, present, and future are evident in many societal symbols, ceremonies, and holidays that serve as powerful reminders that foster social connections and common purpose to enhance collective and individual remembering and resilience. Thus, encouraging pathways for achieving the sense of safety that underlie resilience is a worthwhile endeavor. Pursuing such pathways may be enhanced by encouraging flexibility and a reliance on strengths in order to overcome the barriers that may be encountered along the way.

## Author Contributions

All authors contributed to the writing and editing of this review paper.

## Conflict of Interest

The authors declare that the research was conducted in the absence of any commercial or financial relationships that could be construed as a potential conflict of interest.
